# Unfractionated heparin ameliorates pulmonary microvascular endothelial barrier dysfunction via microtubule stabilization in acute lung injury

**DOI:** 10.1186/s12931-018-0925-6

**Published:** 2018-11-15

**Authors:** Shengtian Mu, Yina Liu, Jing Jiang, Renyu Ding, Xu Li, Xin Li, Xiaochun Ma

**Affiliations:** Department of Intensive Care Unit, The First Affiliated Hospital of China Medical University, No. 92 Bei-er Road, Shenyang, 110001 Liaoning Province People’s Republic of China

**Keywords:** ALI, Endothelial permeability, Cell cytoskeleton, Microtubule, Unfractionated heparin, GEF-H1

## Abstract

**Background:**

Endothelial barrier dysfunction is central to the pathogenesis of sepsis-associated acute lung injury (ALI). Microtubule (MT) dynamics in vascular endothelium are crucial for the regulation of endothelial barrier function. Unfractionated heparin (UFH) possesses various biological activities, such as anti-inflammatory activity and endothelial barrier protection during sepsis.

**Methods:**

Here, we investigated the effects and underlying mechanisms of UFH on lipopolysaccharide (LPS)-induced endothelial barrier dysfunction. C57BL/6 J mice were randomized into vehicle, UFH, LPS and LPS + UFH groups. Intraperitoneal injection of 30 mg/kg LPS was used to induce sepsis. Mice in the LPS + UFH group received intravenous UFH 0.5 h prior to LPS injection. Human pulmonary microvascular endothelial cells (HPMECs) were cultured for analyzing the effects of UFH on LPS-induced and nocodazole-induced hyperpermeability, F-actin remodeling, and LPS-induced p38 MAPK activation.

**Results:**

UFH pretreatment significantly attenuated LPS-induced pulmonary histopathological changes, and increased the lung W/D ratio and Evans blue accumulation in vivo. Both in vivo and in vitro studies showed that UFH pretreatment blocked the LPS-induced increase in guanine nucleotide exchange factor (GEF-H1) expression and myosin phosphatase target subunit 1 (MYPT1) phosphorylation, and microtubule (MT) disassembly in LPS-induced ALI mouse model and human pulmonary microvascular endothelial cells (HPMECs). These results suggested that UFH ameliorated LPS-induced endothelial barrier dysfunction by inhibiting MT disassembly and GEF-H1 expression. In addition, UFH attenuated LPS-induced hyperpermeability of HPMECs and F-actin remodeling. In vitro, UFH pretreatment inhibited LPS-induced increase in monomeric tubulin expression and decrease in tubulin polymerization and acetylation. Meanwhile, UFH ameliorates nocodazole-induced MTs disassembly and endothelial barrier dysfunction.Additionally, UFH decreased p38 phosphorylation and activation, which was similar to the effect of the p38 MAPK inhibitor, SB203580.

**Conclusions:**

UFH exert its protective effects on pulmonary microvascular endothelial barrier dysfunction via microtubule stabilization and is associated with the p38 MAPK pathway.

## Background

Patients with sepsis commonly suffer from abnormal activation of the coagulation system, including diffuse intravascular coagulation and microthrombi formation. Anti-coagulant-mediated regulation of thrombosis-dependent endothelial injury and repair following sepsis may represent a therapeutic approach for patients with sepsis [1]. Sepsis-associated acute lung injury (ALI) is a common and severe consequence of infection, which contributes to significant morbidity and mortality in critically-ill patients [2]. A large number of studies show that lung endothelial cells are key modulators and orchestrators of ALI [3]. Lipopolysaccharide (LPS), the main component of the outer membrane of Gram-negative bacteria, can cause sepsis-associated ALI and endothelial barrier dysfunction [4].

Endothelial permeability is controlled by dynamic cytoskeletal remodeling and inter-cell junction protein complexes, which are also tightly linked to the cytoskeleton [5]. Numerous studies demonstrated that the actin cytoskeleton is critical for controlling endothelial barrier function and vascular permeability. Rho GTPases are the foremost mediators of regulation of actin remodeling, which is involved in endothelial barrier stability in response to different stimuli [6–8]. The Rho guanine nucleotide exchange factor, GEF-H1, acts as a microtubule-dependent regulator and couples microtubules to the Rho GTPase-dependent actin cytoskeleton [9]. An increasing body of evidence corroborates the role of microtubule dynamics in regulation of endothelial permeability [10, 11].

Unfractionated heparin (UFH), a widely used anticoagulant drug, participates in the regulation of multifarious biological functions [12]. We previously demonstrated that LPS induced remodeling of the F-actin cytoskeleton and formation of stress fibers, which increased human umbilical vascular endothelial cell (HUVEC) permeability; in contrast, UFH protected cells from endothelial hyperpermeability [13]. Furthermore, we also showed that UFH attenuated pulmonary vascular hyperpermeability in mouse model of sepsis via the RhoA/Rho kinase (ROCK) pathway [14]; however, the mechanism of action was not clear. Therefore, we investigated whether UFH ameliorates LPS-induced endothelial barrier dysfunction by inhibiting MT disassembly, and regulating GEF-H1 expression and F-actin remodeling.

The p38 MAPK pathway is involved in various biological functions, including endothelial barrier function in response to exogenous and endogenous stimuli [15, 16]. We observed that UFH regulated anti-inflammatory activity by inhibiting p38 MAPK activation [17, 18]. Hence, we investigated whether the p38 MAPK pathway was involved in UFH-mediated attenuation of LPS-induced MT disassembly and endothelial barrier dysfunction.

## Methods

### Reagents and antibodies

LPS from *Escherichia coli* 055:B5 was obtained from Sigma (St. Louis, MO, USA), and UFH was obtained from Shanghai No.1 Biochemical & Pharmaceutical Co. (China). Rabbit polyclonal α-tubulin, GEF-H1, p-MYPT1, p38 MAPK, and p-p38 MAPK antibodies were purchased from Cell Signaling Technology (Beverly, MA, USA). Mouse monoclonal acetylated-α-tubulin and the p38/MAPK inhibitor SB203580 were obtained from Abcam (Cambridge, MA, USA). Nocodazole was purchased from Sigma-Aldrich (St. Louis, MO). Mouse monoclonal antibody against GAPDH was from Invitrogen (Carlsbad, CA, USA). Tetraethyl rhodamine isothiocyanate (TRITC)-phalloidin (Solarbio, China), donkey anti-mouse Cy2 (Jackson, USA), and donkey anti-rabbit Alexa Fluor® 488 (Abcam, USA) were used for immunofluorescence.

### Animal studies

Male C57BL/6 mice weighing 20–25 g were obtained from the Experimental Animal Center of China Medical University. Mice were randomized into four groups: vehicle, UFH, LPS and LPS + UFH. Briefly, mice were anesthetized and given a 30 mg/kg body weight LPS (in 100 μL of saline) intraperitoneally. Subcutaneous injection of 8 units UFH diluted in sterile saline (LPS + UFH group) or equal volume sterile saline (LPS group) was administered 30 min prior to the LPS injection. The dose of LPS and UFH used in vivo are identical to that used previously [14, 19]. After 6 h, the animals were killed under anesthesia and lung tissue samples were collected for subsequent experiments. Evans blue extravasation, lung wet/dry (W/D) weight ratio, and histological assessment of lung injury was conducted as previously described [14]. The animal protocols were formulated in accordance with the guidelines of our University Experimental Animal Administration Committee.

### Cells and cell treatment

HPMECs were supplied by ScienCell Research Laboratories (USA). HPMECs were grown in Dulbecco’s modified Eagle’s medium (DMEM) containing 10% fetal bovine serum (Invitrogen, USA), and cultured at 37 °C in a laboratory CO_2_ incubator with a humidified atmosphere of 5% CO_2_–95% air. HPMECs were utilized at passages 4–8 in all experiments.

### Cell viability assay

Methyl thiazoyltetrazolium (MTT) was adopted to detect the toxicity of LPS on HPMECs. The cells (1–2 × 10^4^ cells/well) were plated on 96-well plates and stimulated with different concentrations LPS for 6 h. Then HPMECs were washed with PBS and incubated with 200 μl MTT medium (1 mg/ml) at 37 °C for 4 h. Afterwards, the medium was discarded and dimethyl sulfoxide (DMSO) (150 μl/well) was used to solubilize formazan. Optical densities (OD) were measured at 490 nm using a plate-reading spectrophotometer. The value of each well was presented as a percentage of the control group. The experiments were repeated five times and the data were calculated as means ± SD.

### Measurement of transendothelial permeability

HPMECs (1.5 × 10^5^ cells/well) were seeded on the 24-well Transwell system (Greiner Bio-One, 0.4-mm pore size, 6.5-mm diameter, transparent, Costar,The Netherlands). The cells were cultured for 4 days and the media was replaced with serum-starved medium for 2 h before exposure to any inhibitor/stimulator. The endothelial cell barrier function was evaluated by measurements of transendothelial electrical resistance (TEER) across confluent cells utilizing Millicell-ERS (MERS00002, Millipore, Bedford,USA). Transendothelial permeability was analyzed using Chemicon’s in vitro vascular permeability assay, which utilizes fluorescein isothiocyanate–dextran (FITC-dextran, 40-kDa, Sigma, USA) [20]. The Transwell plate was removed from the incubator to quantify the passage of FITC-dextran across the monolayer. Using a micropipetter, media (50 μl) was removed from the bottom chamber of each Transwell and transferred to a 96-well plate. The 96-well fluorescence plate reader was used at an excitation wavelength of 488 nm and emission wavelength of 515 nm. Data was represented graphically as raw data in the arbitrary fluorescence units generated by the plate reader.

### Immunofluorescence and image analysis

HPMECs were seeded on glass coverslips and maintained as described above. The cells were then fixed with 4% paraformaldehyde for 30 min, permeabilized with 0.1% Triton X-100 for 5 min, washed in phosphate buffered saline (PBS), and blocked with 5% bovine serum albumin (BSA) for 1 h. The coverslips were incubated with rabbit anti-α-tubulin (1:100) or mouse anti-acetylated-α-tubulin primary antibody (1:200) at 4 °C overnight for determining the MT and acetylated tubulin structure. The coverslips were washed with PBS, followed by incubation with secondary antibodies for 1 h at 37 °C. The actin cytoskeleton was examined by immunofluorescence staining with TRITC-phalloidin for 30 min at 37 °C. The nuclei of the HPMECs were counterstained by DAPI. HPMECs were imaged by microscopy (Olympus BX61, Tokyo, Japan). Images obtained using the 60× objective were processed using the Image J software (National Institute of Health, Bethesda, MD) to outline the cell borders and compute the unoccupied area [13].

### Extraction and quantification of tubulin fractions

The monomeric and polymerized tubulin fractions were extracted from HPMECs using a method previously described by Putnam [21]. HPMECs cultured in 6-well culture plates were washed in an MT stabilization buffer (MTSB, 37 °C). MTSB contained 0.1 M piperazine-1, 4-bisethanesulfonic acid (PIPES), 2 mM ethylene glycol-bis (β-aminoethylether)-N,N,N9,N9-tetraacetic acid (EGTA), 0.1 mM ethylene diamine tetraacetic acid (EDTA), 0.5 mM MgCl_2_, and 20% glycerol (pH 6.8). HPMECs were incubated with MTSB + 0.1% Triton X-100 + protease inhibitor cocktail (1:100, Biotool) + phenylmethylsulphonyl fluoride (PMSF, 1:100, Beyotime Biotechnology) + phosphatase inhibitor cocktail (1:100, Biotool). The monomeric and polymeric tubulin fractions were quantified by western blot analysis.

### Western blot analysis

The monomeric and polymeric tubulin fractions were prepared as described above. The protein extracts were probed using rabbit anti-α-tubulin antibody (1:1000). Equal amounts of protein were separated by sodium dodecyl sulfate-polyacrylamide gel electrophoresis (SDS-PAGE) and electrotransferred to polyvinylidene fluoride (PVDF) membranes. Then, the membranes were blocked for 1 h in 5% BSA in Tris-buffered saline Tween 20 (TBST) and incubated overnight with primary antibodies against acetylated tubulin (1:2000), GEF-H1 (1:1000), p-MYPT1 (1:1000), p38 (1:1000), and p-P38 (1:1000). After washing with TBST, the membranes were incubated with horseradish peroxidase conjugated goat anti-rabbit or goat anti-mouse IgG (Bio-Rad). GAPDH was reprobed as a loading control. The immunocomplexes were developed using the enhanced chemiluminescence (ECL) Plus kit (Amersham). The Image J software (NIH) was used to quantify the intensity values of the bands. Each experiment was repeated thrice.

### Statistical analysis

All statistical analyses were performed using Graphpad Prism 6.0 software. The results were expressed as mean ± SD. Statistical analyses were conducted using one-way ANOVA followed by post hoc analysis. *P* < 0.05 indicated a statistical significance.

## Results

### UFH ameliorates LPS-induced endothelial barrier dysfunction by inhibiting MT disassembly and dynamics of GEF-H1 in vivo

**Fig. 1:**
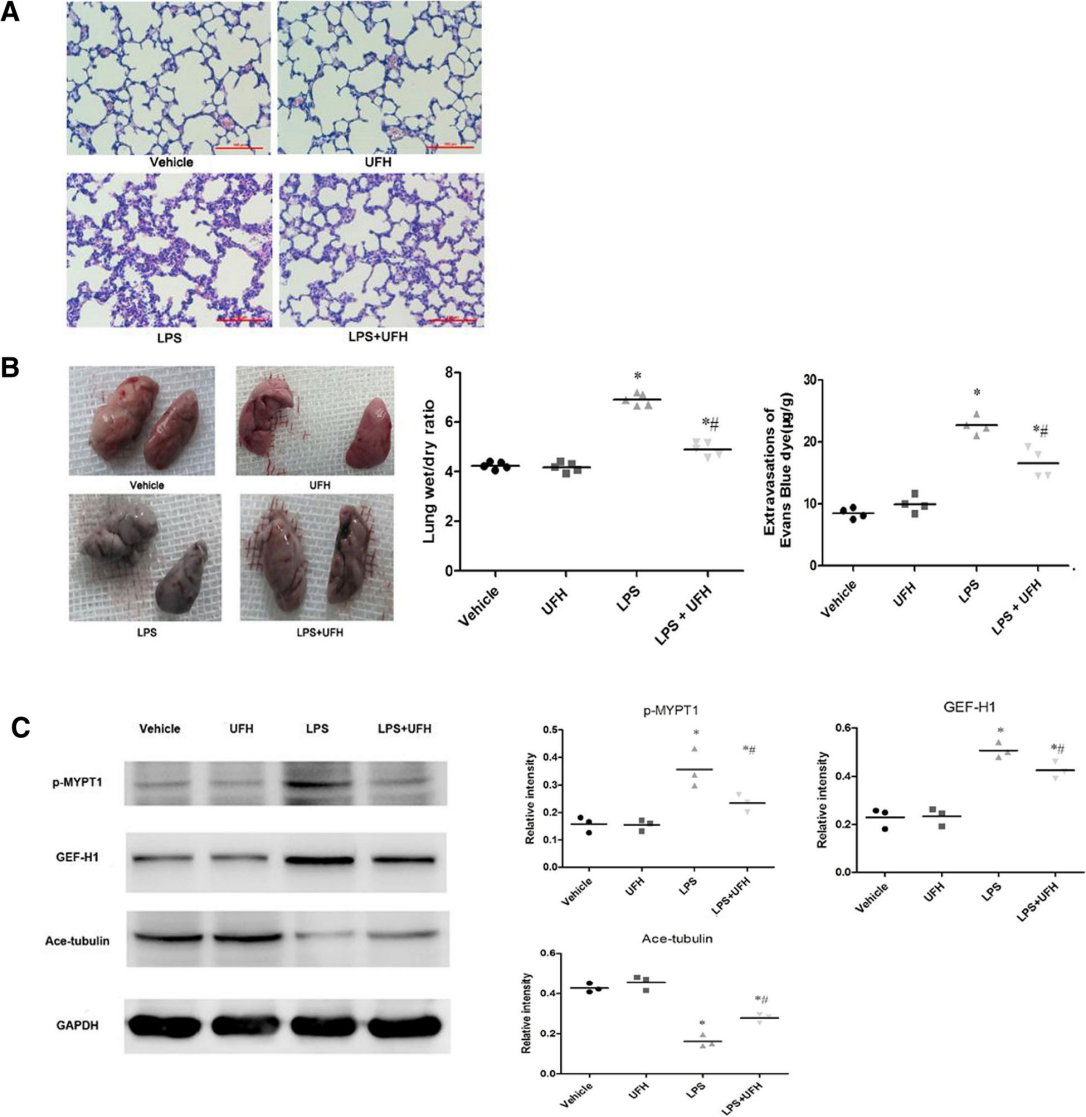
UFH ameliorates LPS-induced endothelial barrier dysfunction by inhibiting MT disassembly and dynamics of GEF-H1 in vivo. C57BL/6 J mice were challenged with LPS (30 mg/kg, intraperitoneally) for 6 h with or without subcutaneous injection of UFH (8 U, subcutaneously). **a** HE staining (scale bars = 100 μm). Administration of UFH suppressed LPS-stimulated pulmonary histopathological changes. **b** Lung W/D and lung Evans blue values. LPS-stimulated group show marked increase in the lung W/D ratio and enhanced pulmonary microvascular leakage. **c** Western blot analysis was used to evaluate the levels of acetylated tubulin, GEF-H1, and MYPT1 phosphorylation. Equal protein loading was confirmed using GAPDH. UFH decreased the LPS-stimulated increase in GEF-H1 expression and MYPT1 phosphorylation. Data are presented as means ± SD (*n* = 3–5 mice per group). t-test, *P < 0.05, compared to vehicle. t-test, # P < 0.05, compared to LPS alone

Hematoxylin and eosin (HE) staining was performed to assess the effects of heparin on the magnitude of lung injury. As expected, pulmonary tissue sections in the vehicle- and UFH-treated mice showed typical structure and clear air sacs under the microscope. In contrast, the lungs of LPS-stimulated animals exhibited obvious neutrophil infiltration and hemorrhage, alveolus collapse, and extensive alveolar septum thickening. However, administration of UFH suppressed the LPS-stimulated pulmonary histopathological changes (Fig. 1a). These findings implied that UFH protected mice against LPS-induced lung injury.

The lung W/D ratio was detected to investigate the effect of heparin on LPS-induced lung edema. The LPS-stimulated group showed a marked increase in the lung W/D ratio (Fig. 1b). Samples were dyed with Evans blue to evaluate pulmonary microvascular leakage after lung injury [14], and Evans blue accumulation (EBA) was observed in the LPS-stimulated group (Fig. 1b). However, UFH pretreatment dramatically ameliorated the changes related to LPS-stimulated lung edema and pulmonary microvascular leakage. In some instances, lungs may not have been completely perfused prior to EBA assay (Fig. 1b), which could impact on interpretation of EBA scores as an accurate indicator of vascular injury. Nevertheless, these results implied the protective effect of UFH against LPS-induced lung vascular dysfunction.

Western blot analysis was performed to further assess the expression of MT-related proteins in ALI mice. Compared to the vehicle group, the LPS group showed decrease in the level of acetylated tubulins, which represent a pool of stable MTs. However, UFH markedly ameliorated the LPS-stimulated decrease in acetylated tubulin levels. Moreover, UFH also decreased the LPS-stimulated increase in GEF-H1 expression and MYPT1 phosphorylation (Fig. 1c). These results demonstrate that UFH may ameliorate LPS-induced pulmonary microvascular endothelial barrier dysfunction though microtubule stabilization and inhibition of GEF-H1. However, the mechanism underlying these effects requires further investigations.

### Effects of LPS on cell viability and permeability

**Fig. 2:**
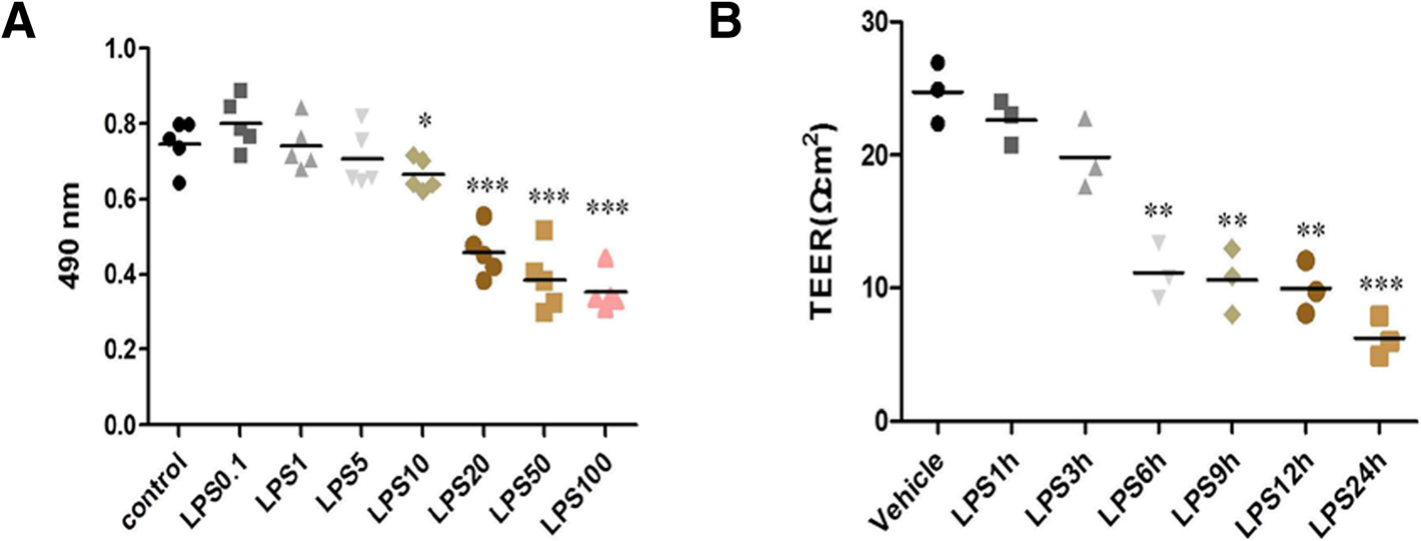
Effect of LPS on cell viability and permeability. **a** Cell viability was measured using the cell viability assay after stimulation with different concentrations LPS (0.1 μg/ml, 1 μg /ml, 5 μg/ml, 10 μg/ml, 20 μg/ml, 50 μg/ml, or 100 μg/ml) for 24 h. The viability of HPMECs was slightly reduced by 10 μg/ml LPS and significantly decreased by ≥20 μg/ml LPS. **b** Cell permeability was measured by the TEER across HPMECs after LPS (10 μg/ml) stimulation for different time periods (1, 3, 6, 9, 12, or 24 h). HPMECs stimulated by LPS for more than 6 h showed marked decrease in the TEER of HPMEC monolayer. Values are means ± SD of five independent experiments. t-test, * *P* < 0.05 compared to the vehicle group. t-test, ** *P* < 0.01 compared to the vehicle group. t-test, *** *P* < 0.001 compared to the vehicle group

Since cell viability is the most direct indicator of cell state, the effect of LPS on HPMEC viability was assessed using the MTT assay. No significant difference in cell viability was observed between HPMECs stimulated with LPS (0.1–5 μg/ml) and the control group. However, HPMEC viability was slightly reduced by treatment with 10 μg/ml LPS, and significantly decreased by treatment with LPS > 20 μg/ml (Fig. 2a). Therefore, we selected 10 μg/ml LPS for stimulating cells in subsequent experiments.

TEER was used to determine the permeability of the HPMEC monolayer after LPS (10 μg/ml) stimulation for different time periods. We observed that LPS decreased the permeability of HPMECs in a time-dependent manner. HPMECs stimulated by LPS for more than 6 h showed marked decrease in the monolayer TEER (Fig. 2b). Thus, HPMEC stimulation by 10 μg/ml LPS for 6 h was selected as conditions for subsequent experiments.

### UFH attenuates LPS-induced hyperpermeability of HPMECs and F-actin remodeling

**Fig. 3:**
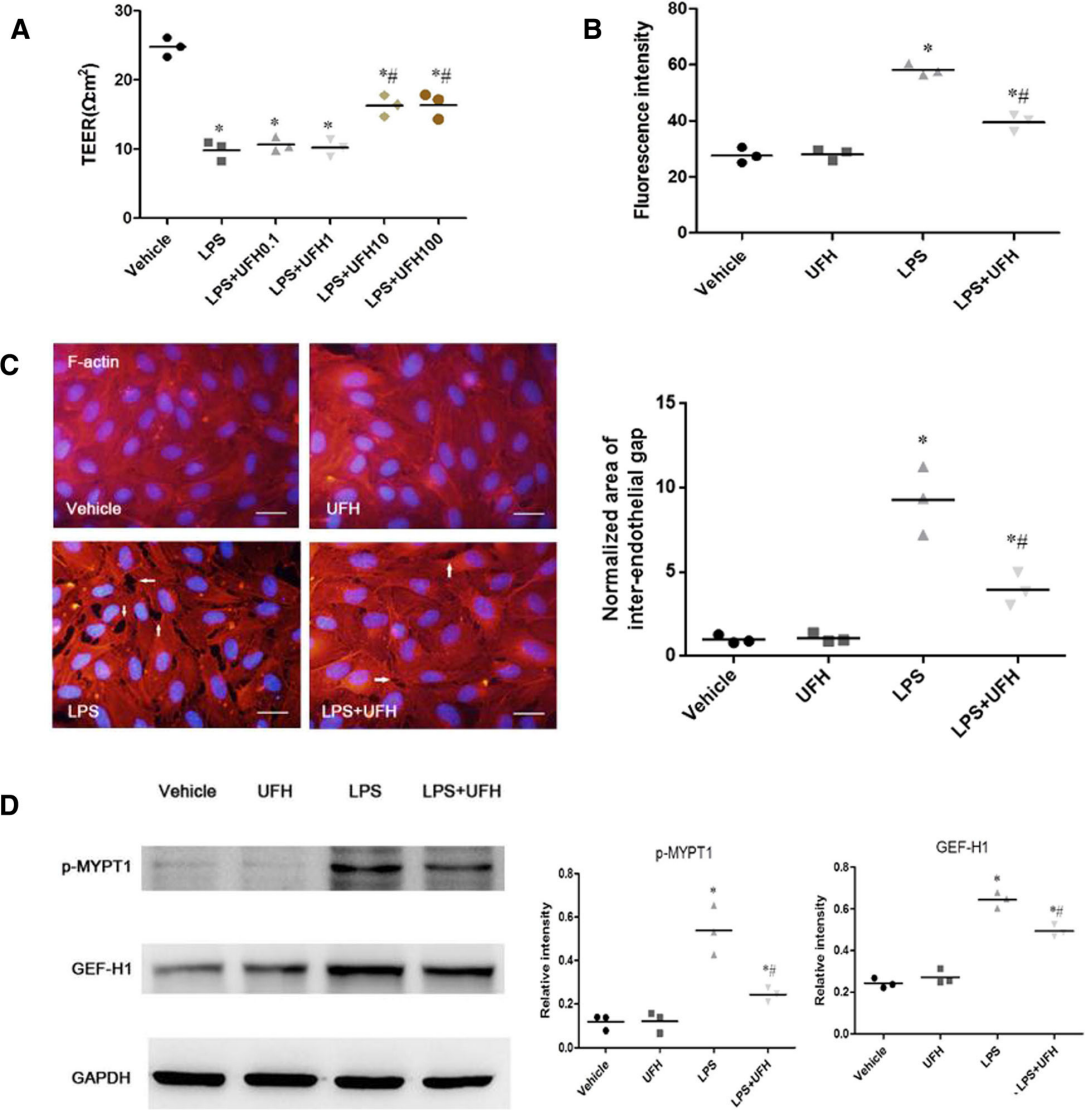
Effect of UFH pretreatment on LPS-induced increase in endothelial cell permeability and F-actin remodeling. HPMECs were pretreated with vehicle or UFH, followed by LPS stimulation for 6 h. **a** The TEER across the cells were measured. UFH (≥ 10 U/ml) significantly protected cells from LPS-stimulated endothelial barrier dysfunction. **b** The influx of FITC-conjugated dextran across cells was determined. UFH (10 U/ml) pretreatment decreased LPS-stimulated hyperpermeability. **c** Actin cytoskeletal remodeling and area of inter-endothelial gaps. Actin cytoskeletal remodeling was examined by immunofluorescence staining with TRITC-phalloidin. Arrows indicate intercellular gaps. Vehicle and UFH group displayed intact monolayer, and stress fibers were rarely observed. UFH pretreatment decreased LPS-induced stress fibers and intercellular gaps (scale bars = 50 μm). The area of the gaps in the microscope fields was assessed and normalized to the area of gaps induced with LPS alone treatment. UFH pretreatment prevented the LPS-induced changes in the actin cytoskeleton. For each group, nine microscope fields from three parallel experiments were analyzed. **d** Western blot analysis was used to evaluate GEF-H1 levels and MYPT1 phosphorylation. Equal protein loading was confirmed by GAPDH levels. Data are representative of three independent experiments. t-test, * *P* < 0.05 compared to the vehicle group; t-test, # P < 0.05 compared to the LPS-treated group

To assess the effect of UFH on the LPS-induced hyperpermeability of HPMECs, different doses of UFH (0.1 U/ml, 1 U/ml, 10 U/ml, and 100 U/ml) were administrated 30 min prior to LPS injection, and the permeability of HPMEC monolayer was assessed by measuring TEER across cells. Apparently, 10 U/ml or more UFH significantly inhibited LPS-stimulated endothelial barrier dysfunction (Fig. 3a). Therefore, administration of 10 U/ml UFH 30 min prior to LPS injection was selected as the condition for subsequent experiments. Next, we detected the influx of FITC-conjugated dextran to assess the permeability of HPMECs. UFH (10 U/ml) pretreatment also decreased LPS-stimulated hyperpermeability (Fig. 3b).

We performed an immunofluorescence assay to investigate whether UFH pretreatment affects the actin cytoskeleton. Stress fibers were not formed in quiescent HPMECs and UFH-treated cells. However, 6 h LPS exposure caused F-actin remodeling, including the formation of stress fibers and paracellular gaps. In contrast, UFH pretreatment prevented the LPS-induced changes in the actin cytoskeleton structure (Fig. 3c).

Next, we assessed changes in the levels of related proteins, GEF-H1 and p-MYPT1, by western blot analysis. GEF-H1 is important for Rho GTPase activation, MYPT1 phosphorylation, actin remodeling, and endothelial barrier dysfunction [22]. Our study showed that LPS-stimulation of HPMECs significantly increased GEF-H1 expression and MYPT1 phosphorylation, which were remarkably decreased by UFH pretreatment (Fig. 3d) and was consistent with the results of our animal study. These findings indicated the mechanism of UFH pretreatment-mediated protection against LPS-induced hyperpermeability of HPMECs and F-actin remodeling.

### UFH ameliorates LPS-induced endothelial barrier dysfunction by inhibiting MT disassembly

**Fig. 4:**
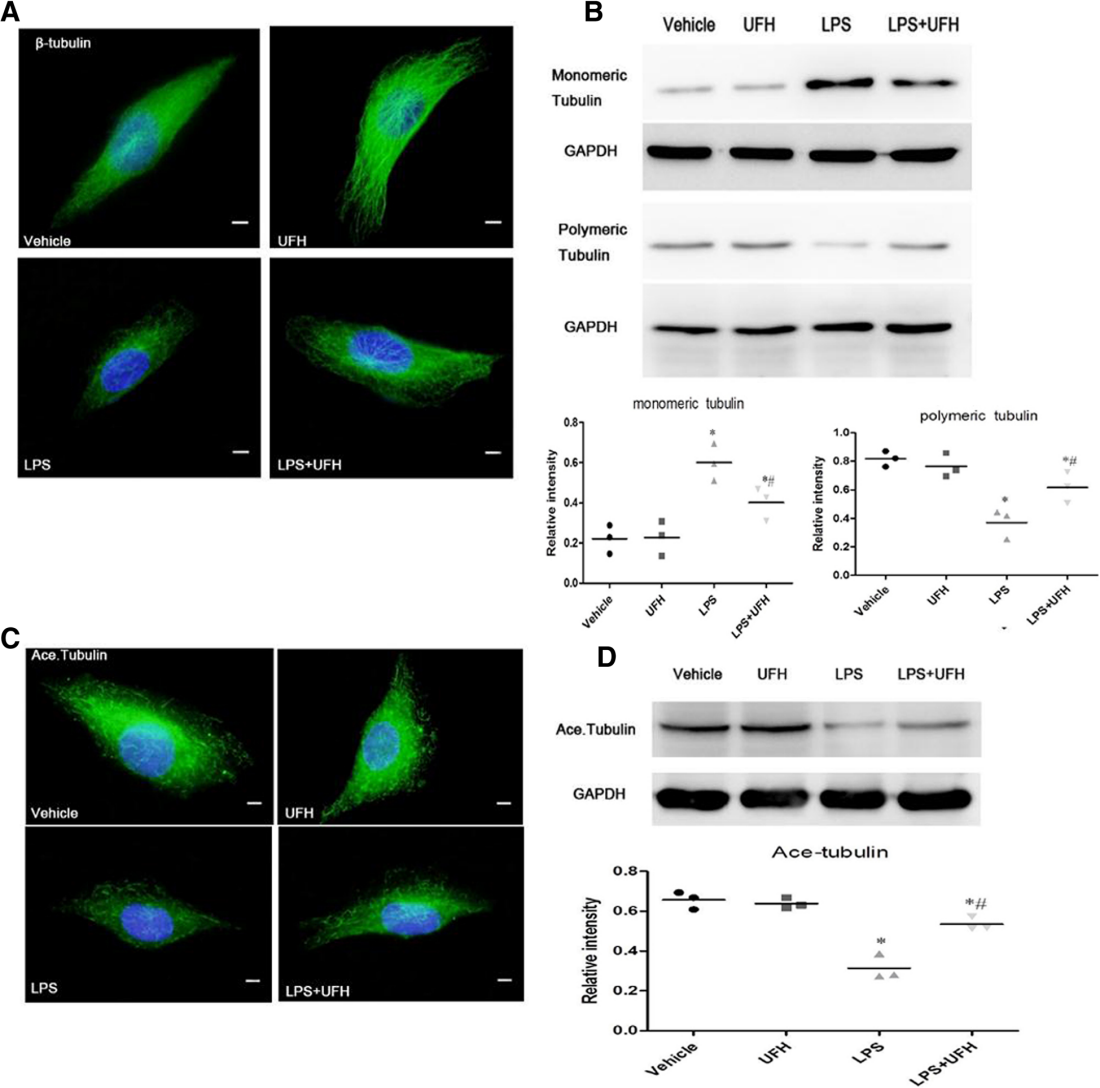
Effect of UFH pretreatment on LPS-induced disassembly of MTs. HPMECs were pretreated with vehicle or UFH (10 U/ml, 30 min) followed by LPS (10 μg/ml) stimulation for 6 h. **a** MT remodeling was examined by immunofluorescence staining with Alexa Fluor® 488, (× 60, scale bars = 10 μm). LPS stimulation triggered striking MT disassembly, which was prevented by UFH pretreatment. **b** Western blotting was used to detect polymeric/monomeric tubulin ratio. GAPDH was used as the internal control for monomeric and polymeric tubulin. UFH pretreatment decreased monomeric tubulin content and increased tubulin polymerization. **c** Acetylated tubulin was examined by immunofluorescence staining with cyanine 2, (× 60, scale bars = 10 μm). UFH increased the tubulin acetylation in HPMECs treated with LPS. **d** Pool of stable MTs was determined by western blot analysis with antibodies against acetylated tubulin. UFH pretreatment inhibited the LPS-induced decrease in acetylated tubulin level. Data are representative of three independent experiments and are shown as mean ± SD. t-test, * *P* < 0.05 compared to the vehicle group. t-test, # P < 0.05 compared to the LPS-treated group

MTs play an essential role in the regulation of endothelial barrier function. MT disassembly stimulates Rho/ROCK activation via GEF-H1, resulting in F-actin remodeling and loss of endothelial barrier. Immunofluorescence was used to detect LPS and UFH pretreatment-induced changes in MTs. As shown in Fig. 4a, MTs organized into a faint and uniformly distributed lattice network in quiescent HPMECs and UFH-treated cells. In contrast, LPS stimulation triggered MTs disassembly, whereas UFH pretreatment inhibited these changes.

Furthermore, the monomeric and polymeric tubulin fractions were separated and quantitatively analyzed by western blotting to determine the structural changes of MTs (Fig. 4b). Tubulin existed maximally in the polymeric form in vehicle- and UFH-treated HPMECs. On the contrary, LPS-stimulated HPMECs showed MT disassembly, as indicated by increase in monomeric tubulin content and simultaneous decrease in polymeric tubulin levels. However, MT structure was preserved in UFH-pretreated LPS-stimulated cells.

Since acetylated tubulin contributes to microtubule stabilization, we used immunofluorescence to further confirm the effect of UFH on MT structure [23]. Compared to the vehicle- and UFH-treated cells, HPMEC exposure to LPS remarkably reduced the level of acetylated tubulin (Fig. 4c). However, UFH pretreated LPS-challenged HPMECs showed increase in tubulin acetylation. Furthermore, we detected acetylated tubulin levels by quantitative analysis of western blotting. As shown in Fig. 4d, UFH pretreatment inhibited the LPS-induced decrease in acetylated tubulin levels.

Taken together, these results highlight that UFH participates in modulation of MT organization, which is consistent with the MT assembly completion. The activation state of GEF-H1 depends on its association with MTs. The above observations showed that UFH ameliorated LPS-induced endothelial barrier dysfunction by inhibiting MT disassembly and GEF-H1 expression, thereby resulting in actin remodeling and endothelial barrier dysfunction.

### Effects of UFH on LPS-induced p38 MAPK activation

**Fig. 5:**
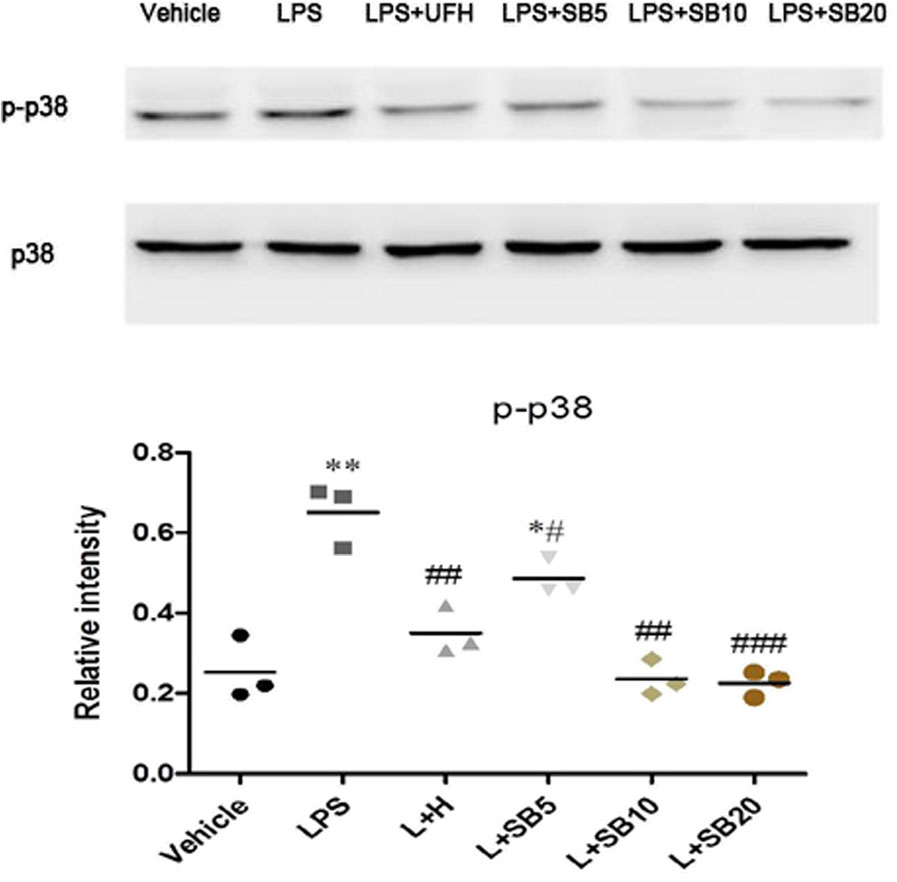
Effects of UFH on LPS-induced p38 MAPK activation in HPMECs. Cells were pretreated with UFH (10 U/ml) or the indicated concentrations of SB203580 (5 μM, 10 μM, and 20 μM) for 30 min, and were then exposed to LPS (10 μg/ml) for 6 h. p-p38 and p38 were detected by western blot analysis. The intensity of the band corresponding to p-p38 was corrected to that of total p38 MAPK. UFH or the p38 MAPK inhibitor SB203580 (10–20 μM) significantly inhibited LPS-induced phosphorylation of p38 MAPK. t-test, * *P* < 0.05, compared to the vehicle-treated control group. t-test, ** P < 0.01, compared to the vehicle-treated control group. t-test, #P < 0.05, compared to the LPS-treated group. t-test, ##P < 0.01, compared to the LPS-treated group. t-test, ###P < 0.01, compared to the LPS-treated group

The p38 MAPK signal transduction pathway plays crucial roles in various endothelial functions, such as proliferation, migration, and tube formation. Therefore, we assessed the effects of UFH on LPS-induced p38 MAPK activation using western blot analysis. Our results demonstrated that LPS-stimulation activated p38 MAPK in HPMECs. However, UFH or the p38 MAPK inhibitor, SB203580 (10–20 μM), significantly inhibited LPS-induced phosphorylation of p38 MAPK (Fig. 5). As inhibition by 10 μM SB203580 was not significantly different from that by 20 μM SB203580, SB203580 (10 μM) was selected for use in subsequent experiments.

### p38 MAPK inhibition represents one of the mechanisms via which UFH ameliorates LPS-induced endothelial barrier dysfunction and causes F-actin remodeling

**Fig. 6:**
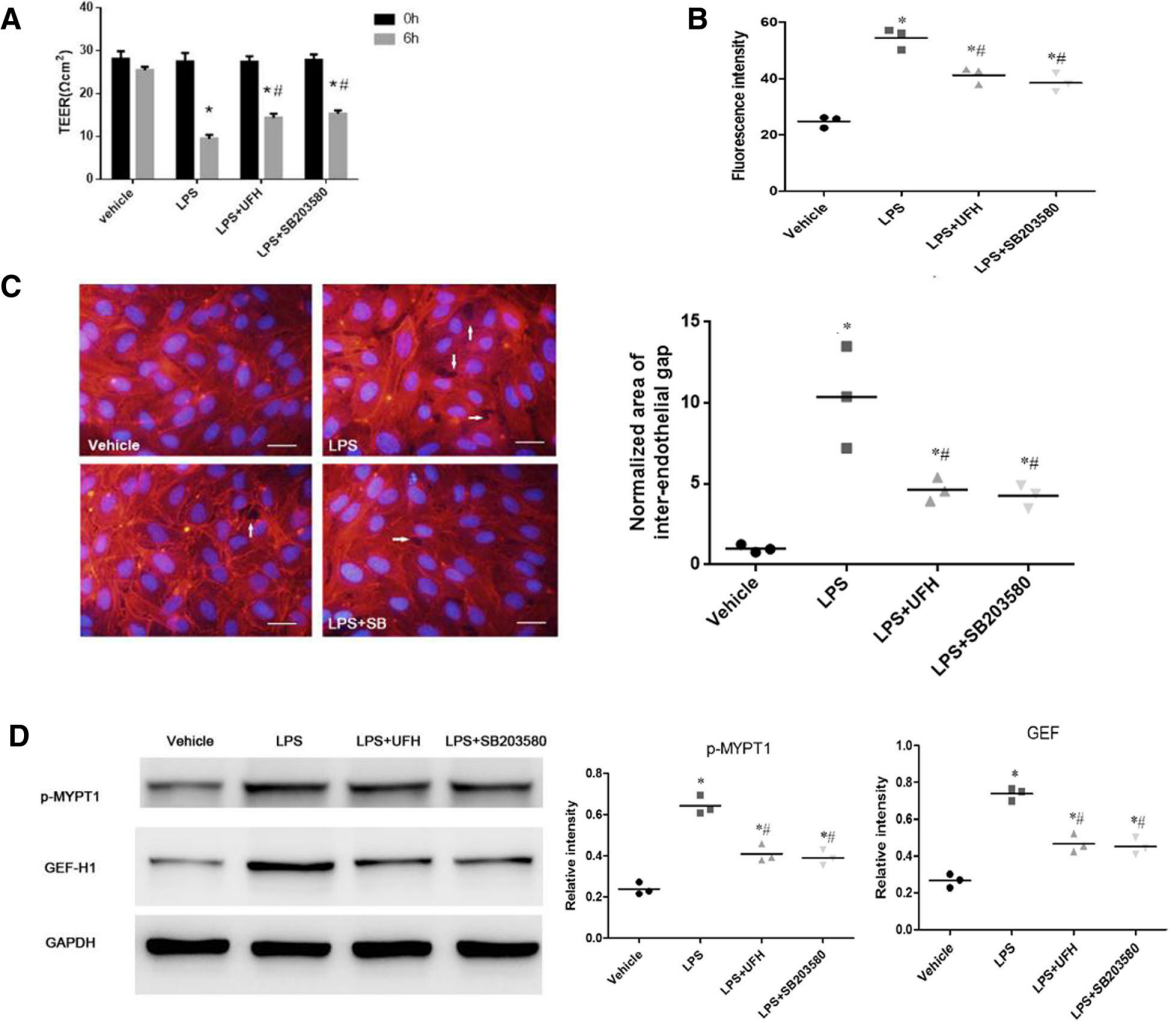
Effect of UFH or the p38 MAPK inhibitor SB203580 pretreatment on LPS-induced increase in endothelial cell permeability and F-actin remodeling. HPMECs were pretreated with UFH (10 U/ml) or SB203580 (10 μM) for 30 min, followed by LPS (10 μg/ml) stimulation for 6 h. **a** The TEER across the cells were measured. Both UFH and SB203580 protected cells from LPS-induced endothelial barrier dysfunction by increasing TEER of the HPMEC monolayer. **b** The influx of FITC-conjugated dextran across the cells was measured. Both UFH and SB203580 protected cells from LPS-induced endothelial barrier dysfunction by decreasing FITC-conjugated dextran leakage. **c** Actin cytoskeletal remodeling and area of inter-endothelial gaps. Actin cytoskeletal remodeling was examined by immunofluorescence staining with TRITC-phalloidin. Arrows indicate intercellular gaps. UFH or SB203580 pretreatment decreased LPS-induced formation of stress fibers (scale bars = 50 μm). The area of gaps in the microscope fields was assessed and normalized to the area of gaps induced with LPS alone. UFH and SB203580 prevented paracellular gaps induced by LPS. Nine microscope fields from three parallel experiments were analyzed for each group. **d** Western blot analysis was used to evaluate the levels of GEF-H1 and MYPT1 phosphorylation. Equal protein loading was confirmed using GAPDH. LPS-induced GEF-H1 expression and MYPT1 phosphorylation were attenuated by UFH as well as SB203580. Data are representative of three independent experiments. t-test, * P < 0.05 compared to the vehicle group, t-test, # P < 0.05 compared to the LPS-treated group

We further studied the association between p38 MAPK inhibition and UFH-mediated amelioration of LPS-induced endothelial barrier dysfunction. Both UFH and SB203580 protected cells from LPS-induced endothelial barrier dysfunction by increasing TEER of HPMEC monolayer and decreasing FITC-conjugated dextran leakage (Fig. 6a and b). Morphological assessment showed that UFH and SB203580 prevented remodeling of actin and paracellular gaps induced by LPS (Fig. 6c). Furthermore, the LPS-induced increase in GEF-H1 expression and MYPT1 phosphorylation were attenuated by both UFH and SB203580 (Fig. 6d). These results indicated that UFH-mediated amelioration of LPS-induced endothelial barrier dysfunction may be related to the p38 MAPK pathway.

### UFH-mediated inhibition of LPS-induced MT depolymerization is related to the p38 MAPK pathway

**Fig. 7:**
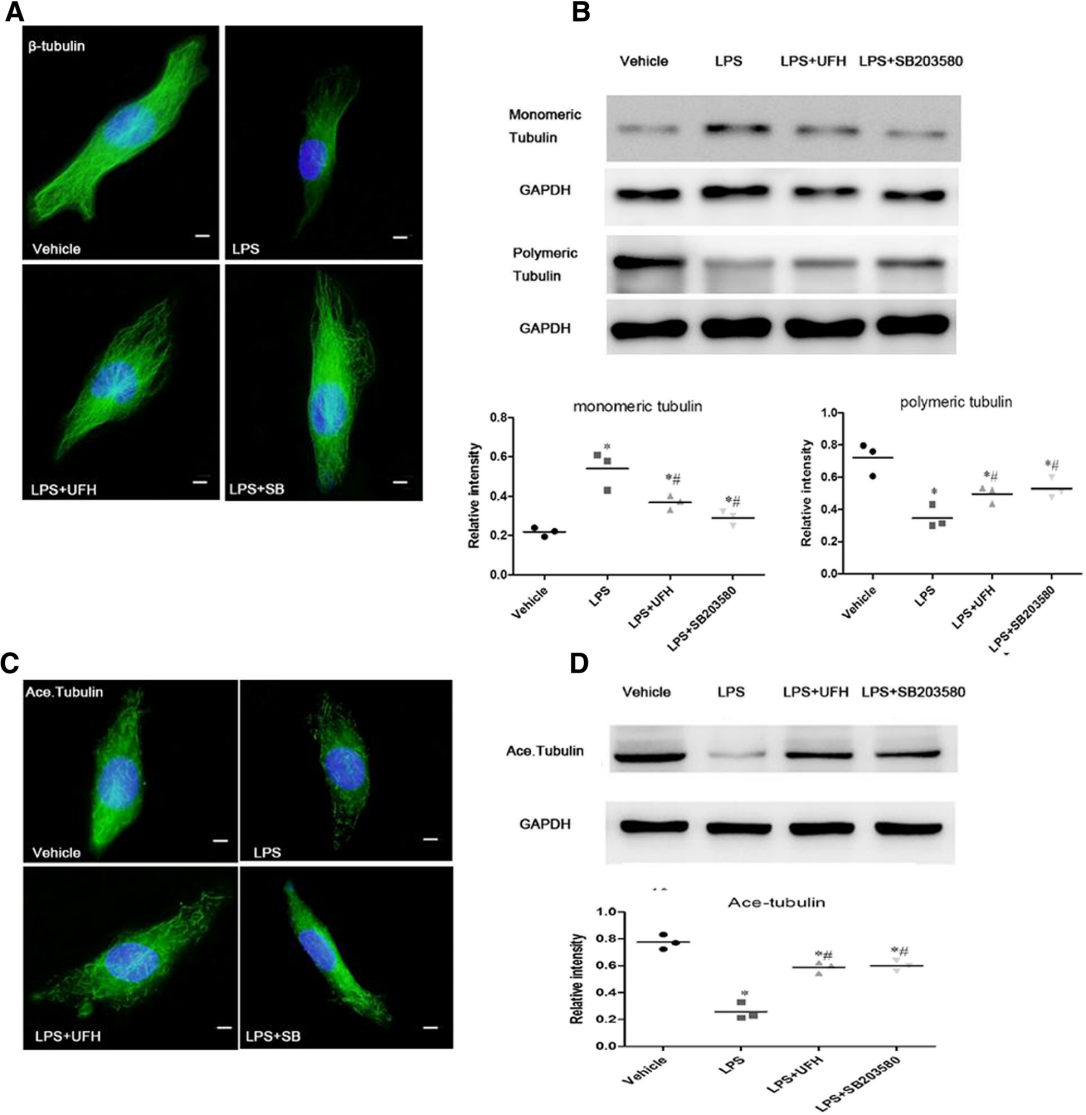
Effect of UFH or SB203580 pretreatment on LPS-induced disassembly of MTs. HPMECs were pretreated with UFH (10 U/ml) or SB203580 (10 μM) for 30 min, followed by LPS (10 μg/ml) stimulation for 6 h. **a** MT remodeling was examined by immunofluorescence staining with Alexa Fluor® 488, (scale bars = 10 μm). UFH and SB203580 prevented LPS-induced MT disassembly. **b** Western blotting was used to detect polymeric/monomeric tubulin. UFH and SB203580 prevented LPS-induced MT disassembly. **c** Acetylated tubulin was examined by immunofluorescence staining with cyanine 2, (scale bars = 10 μm). UFH and SB203580 enhanced acetylated tubulin expression. **d** Pool of stable MTs was determined by western blot analysis with antibodies against acetylated tubulin. UFH and SB203580 enhanced acetylated tubulin levels. Data are representative of three independent experiments and are shown as means ± SD. t-test, * P < 0.05 compared to the vehicle group. t-test, # P < 0.05 compared to the LPS-treated group

Immunofluorescence and western blot analysis were performed to confirm the association of the p38 MAPK pathway with UFH-mediated inhibition of LPS-induced MT depolymerization. We observed that UFH and SB203580 prevented LPS-induced MT disassembly, as demonstrated by tubulin immunofluorescence and western blot analysis of polymeric/monomeric tubulin (Fig. 7a and b). Furthermore, acetylated tubulin was also examined by immunofluorescence and western blot analysis (Fig. 7c and d). Overall, our results indicated that inhibition of p38 MAPK signaling might be one of the mechanisms via which UFH ameliorates LPS-induced endothelial barrier dysfunction by inhibiting MT depolymerization, increasing GEF-H1, and remodeling F-actin.

### UFH ameliorates nocodazole-induced MTs disassembly and endothelial barrier dysfunction

**Fig. 8:**
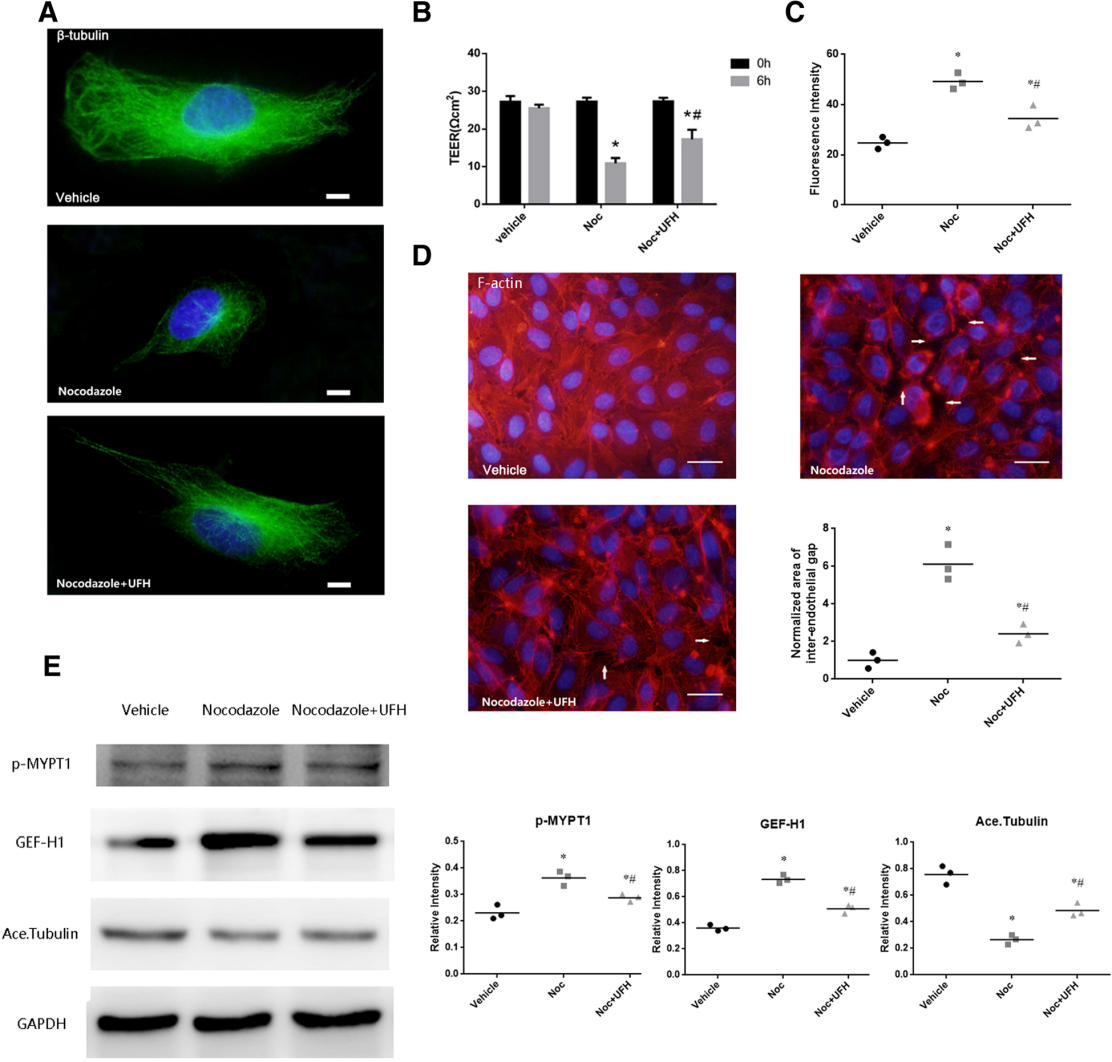
Effect of UFH on nocodazole-induced MTs disassembly and actin cytoskeleton remodeling. HPMECs were pretreated with vehicle or UFH followed by nocodazole (0.2 μM) stimulation for 6 h. **a** MTs remodeling was examined by immunofluorescence staining with Alexa Fluor® 488, (scale bars = 10 μm). **b** The TEER across the cells were measured. **c** The influx of FITC-conjugated dextran across the cells were measured. **d** Actin cytoskeletal remodeling was examined by immunofluorescence staining with TRITC-phalloidin. Arrows indicate intercellular gaps. Vehicle group displayed intact monolayer and stress fibers were rarely observed. UFH pretreatment decreased nocodazole -induced the formation of stress fibers and intercellular gaps, (scale bars = 50 μm). The area of inter-endothelial gaps in the microscope fields was assessed and normalized to the area of gaps induced with nocodazole alone. For each group, total of nine microscope fields from three parallel experiments were analyzed. **e** Western blot analysis was used to evaluate the levels of Acetylated tubulin, GEF-H1 and phosphorylation of MYPT1. Equal protein loading was confirmed by GAPDH antibody. Data are representative of 3 independent experiments. t-test, * P < 0.05 compared to the vehicle group. t-test, # P < 0.05 compared to the nocodazole -treated group

To assess whether MTs disassembly causes endothelial hyperpermeability or F-actin remodeling and the protective effects of UFH are mediated by prevention of microtubule disassembly, HPMECs were pretreated with vehicle or UFH followed by MTs inhibitor nocodazole (0.2 μM) stimulation for 6 h. As shown in Fig. 8a, nocodazole induced MTs disassembly and UFH pretreatment stabilized microtubule. Further analysis showed that nocodazole caused the permeability of HPMECs increased (Fig. 8b) and F-actin remodeling that included the formation of stress fibers and paracellular gaps (Fig. 8c and Fig. 8d). On the contrary, UFH pretreatment attenuated nocodazole-induced endothelial barrier dysfunction and F-actin remodeling (Fig. 8b, c and d). Furthermore, we assessed changes in the levels of related proteins, Acetylated tubulin, GEF-H1 and p-MYPT1, by western blot analysis. Our results demonstrated that nocodazole decreased the acetylation of tubulin, increased the expression of GEF-H1 and MYPT1 phosphorylation, which were all remarkably ameliorated by pretreatment (Fig. 8e). All these results indicated that nocodazole increased the permeability of HPMECs through causing MTs disassembly, increasing GEF-H1 expression and MYPT1 phosphorylation and F-actin remodeling. These results also supported that UFH exerted its protective effects by stabilizing microtubule.

## Discussion

Our study elucidates a novel mechanism via which UFH exerts protective effects in endothelial barrier dysfunction of ALI. This mechanism suggests that UFH protects the endothelial barrier by stabilizing MTs, inhibiting GEF-H1, and remodeling actin in vitro and in vivo, which may be related to its inhibition of LPS-induced activation of p38 MAPK. This provides a new theoretical basis for the use of UFH in the treatment of sepsis-associated ALI in the clinic.

UFH possesses several biological properties beyond its anticoagulant activity, and has been reported to produce clinical benefit in patients with sepsis [24, 25]. Our previous study demonstrated that pretreatment of mice with sepsis with UFH strongly ameliorates inflammation and coagulation [19]. Sepsis is a common disease and is often complicated with ALI. Treatment with UFH can also attenuate inflammatory responses in a rat model of LPS-induced ALI by downregulating nuclear factor-κB signaling pathway [26]. As for the dose of LPS used to study sub-lethal lung injury, a variety of different dosages of LPS have been used, such as 1 mg/kg, 5 mg/kg, 10 mg/kg, 15 mg/kg, 30 mg/kg and 40 mg/kg [27–31]. Low doses of LPS may not be sufficient to induce injury, while high doses may be lethal. In our study, the dose of LPS used was 30 mg/kg, which was referred to previous studies from Kawasaki et al. [30] and Han et al. [14]. An in vivo study showed that UFH pretreatment by inhalation inhibits alveolar procoagulant reaction and early inflammatory response, promotes fibrinolysis, and alleviates pulmonary pathology in rats with endotoxemia-induced lung injury, which is especially more effective than administration of UFH after LPS challenge [32]. It was suggested that UFH pre-treatment was more effective on lung injury. Based on this report and the results of our previous studies, UFH was administered before LPS injection in this study. Additionally, our previous in vitro study showed that the levels of Tie2, an endothelial cell-specific tyrosine kinase receptor affecting stable vasculature, was markedly reduced by 6-h LPS stimulation, which showed no obvious difference when compared with the levels obtained under 12-h LPS stimulation [13]. An in vivo study demonstrated that levels of pulmonary vascular endothelial growth factor (VEGF) at different time points after LPS injection (1, 3, 6, and 10 h) were down-regulated, especially at 6 h after LPS treatment [33]. In the present study, we observed that HPMECs stimulated by LPS for more than 6 h showed marked decreased in the TEER of HPMEC monolayer. Based on the above results, we assessed changes in vascular injury at 6 h post-LPS in this study. However, 6 h post-LPS may not be the timepoint of peak injury in mice, and that lack of an injury timecourse is a weakness of the study.

Loss of endothelial barrier integrity is central to the pathogenesis of sepsis-associated ALI [34]. nocodazole, as a microtubule poison, destroys the vascular endothelial barrier [35]. Our results showed that nocodazole increased the permeability of HPMECs, expression of GEF-H1 and MYPT1 phosphorylation and F-actin remodeling, suggesting microtubule disassembly is sufficient to cause the increase in GEF-H1 and p-MYPT1.The microtubule stabilizer, taxol, inhibits thrombin or TGF- β1-induced activation of Rho GTPases in endothelial barrier dysfunction during lung injury [36, 37]. A study showed that GEF-H1 overexpression antagonized the heparin-mediated inhibition of pulmonary artery smooth muscle cell (PASMC) proliferation, indicating that the inhibitory effect of heparin was exerted partially via the GEF-H1/RhoA/ROCK/p27 signaling pathway and GEF-H1 down-regulation [38]. Additionally, a previous report revealed that siRNA-mediated depletion of GEF-H1 in HeLa cells prevents nocodazole-induced microtubule disassembly and RhoA activation and cell contractility were rescued by reintroduction of siRNA-resistant GEF-H1, suggesting GEF-H1 couples nocodazole-induced microtubule disassembly to cell contractility via RhoA [35]. In our study, UFH pretreatment blocked the LPS-induced increase in GEF-H1 expression in LPS-induced ALI mice and HPMECs. Our results indicated that UFH ameliorates LPS-induced endothelial barrier dysfunction by inhibiting GEF-H1 expression. We also confirmed that UFH ameliorates nocodazole-induced MTs disassembly and endothelial barrier dysfunction via increasing the acetylation of tubulin and decreasing the expression of GEF-H1 and MYPT1 phosphorylation, implying the protective effects of UFH can be reversed by downstream activation of microtubule disassembly.

Myosin phosphatase regulates the interaction of actin and myosin in response to small GTPase Rho signaling, and Rho activity inhibits myosin phosphatase via ROCK. MYPT1 is one of the subunits of myosin phosphatase, phosphorylation of which results in cytoskeletal reorganization. In this study, we observed that UFH pretreatment remarkably decreased LPS-induced increase in MYPT1 phosphorylation both in vitro and in vivo. This is in agreement with the results of our previous study, which showed that UFH treatment significantly attenuated the increase in p-MYPT1 levels in the lung tissues of LPS-challenged mice [14].

The Rho-Rho kinase cascade triggers MYPT1 phosphorylation, which might lead to actin reorganization and stress fiber formation, resulting in the formation of paracellular gaps and increase in endothelial permeability. GEF-H1 links changes in microtubule integrity to a Rho-dependent regulation of the actin cytoskeleton. Our previous study showed that pretreatment with UFH decreased the formation of stress fiber and intracellular gaps induced by LPS [13], which is in agreement with the results of this study.

GEF-H1 is uniquely positioned for transducing signals after MT depolymerization. It is bound to MTs and is released and activated upon MT depolymerization. The LPS-induced decrease in dynamic MT pool occurs as a result of posttranslational modifications of tubulin [39]. Further, our study showed that UFH administration increased the levels of polymerized tubulin and decreased monomeric tubulin levels in LPS-challenged cells. Additionally, UFH pretreatment increased acetylated tubulin levels both in vitro and in vivo, which could be the mechanism underlying UFH-mediated prevention of LPS-induced MT instability.

The p38 MAPK pathway plays a crucial role in the regulation of cell growth, differentiation, proliferation, apoptosis, invasion, and metastasis. MTs act as docking sites and reservoirs for various proteins, including MAPKs, PI3Ks, and GTPases. p38 MAPK signaling was affected by microtubule state [40]. SB-203580, a p38 MAPK inhibitor, attenuated nocodazole-induced MT depolymerization, actin remodeling, and endothelial barrier dysfunction [41]. Previous studies showed SB-203580 decreased p38 phosphorylation [42, 43], which was in accordance with our result in this study. However, some studies reported that phosphorylation of p38 MAPK was not influenced by SB 203580 [44, 45]. Therefore, the involvement of p38 still need to be confirmed by depletion experiment using siRNA technique. Zhou et al. showed that the p38/mitogen-activated protein kinase pathway is implicated in LPS-induced microtubule depolymerization via up-regulation of microtubule-associated protein 4(MAP4) phosphorylation in human vascular endothelium; however, SB203580 did not completely block the LPS-induced MAP4 phosphorylation in ECs, suggesting other LPS-related mechanisms are involved in LPS-induced MT disassembly [46]. Sepsis-related inflammatory factors promoted endothelial cell activation, dysfunction, and apoptosis via activation of the p38 MAPK pathway [47]. UFH was reported to enhance the barrier function of resting endothelium [48–50]. An early study has revealed that UFH suppress inflammatory-mediated tissue factor expression and increase the anticoagulant properties of macro- and micro-vascular endothelial cells [51]. Our previous studies showed that UFH exerted its anti-inflammatory effect by inhibiting the p38 MAPK pathway [17, 18], which were in agreement with the results of this study. p38 MAPK, a downstream effectors of RhoA-GTPase signaling, was activated by RhoA [52], while increasing of GEF-H1 expression could lead to the activation of RhoA. The underlying mechanisms of UFH inhibition on p38 MAPK may be related to the inhibition of GEF-H1 expression. In addition, a previous report uncovered that microtubule disassembly by nocodazole induced rapid decreases in transendothelial electrical resistance and actin cytoskeletal remodeling [53], which was consistent with our results. In this study, we observed that UFH prevented MT destabilization caused by LPS-induced actin microfilament cytoskeletal changes and barrier dysfunction in HPMECS, which is similar to the effects of SB 203580 treatment. This indicates that UFH protected cells from LPS-stimulated pulmonary microvascular endothelial dysfunction by stabilizing MTs involved with the p38 MAPK pathway. However, MT dynamics is regulated by other pathways [6, 46, 47], and whether UFU acts in regulating endothelial barrier activity requires further investigation.

It is generally known that sugar-based endovascular structure is essential for proper microvascular physiology, which gets damaged by SIRS mainly due to the oxidative stress [54]. Our previous studies had found that UFH or N-desulfated/re-N-acetylated heparin (NAH) can protect glycocalyx from shedding in septic shock model of beagle dogs or mice [55, 56]. The effect of UFH on endothelial barrier protection in the ALI model may be implicated with building bricks for self-reconstitution of the glycocalyx .

Compared to young mice, aged mice exhibit increased severity of lung injury, as demonstrated by higher diffuse alveolar damage and alveolar wall thickening, and increased alveolar and vascular permeability [57]. Future studies on UFH’s effect on LPS-induced ALI in aged mice are warranted. Meanwhile, confirmation could also be performed in future studies of female mice. In the present study, UFH was administered before LPS injection, which is clinically irrelevant. Therefore, a clinically relevant study, in which UFH will be administered after LPS induction, is still required in future.

## Conclusions

In conclusion, we propose a novel UFH-dependent mechanism of endothelial barrier protection in the ALI model, which proceeds via microtubule stabilization and is associated with the p38 MAPK pathway. Based on these results, UFH is likely a promising therapeutic agent for ameliorating the development of ALI.

## Abbreviations

ALI: Acute lung injury
BSA: Bovine serum albumin
DMEM: Dulbecco’s modified Eagle’s medium
DMSO: Discarded and dimethyl sulfoxide
EBA: Evans blue accumulation
ECL: Enhanced chemiluminescence
EDTA: Ethylene diamine tetraacetic acid
EGTA: Ethylene glycol-bis (β-aminoethylether)-N, N, N9, N9-tetraacetic acid
GEF-H1: Guanine nucleotide exchange factor
HE: Hematoxylin and eosin
HPMECs: Human pulmonary microvascular endothelial cells
HUVEC: Human umbilical vascular endothelial cell
LPS: Lipopolysaccharide
MT: Microtubule
MTSB: MT stabilization buffer
MTT: Methyl thiazoyltetrazolium
MYPT1: Myosin phosphatase target subunit 1
OD: Optical densities
PASMC: Pulmonary artery smooth muscle cell
PBS: Phosphate buffered saline
PIPES: Piperazine-1, 4-bisethanesulfonic acid
PMSF: Phenylmethylsulphonyl fluoride
PVDF: Polyvinylidene fluoride
ROCK: Rho kinase
SDS-PAGE: Sodium dodecyl sulfate-polyacrylamide gel electrophoresis
TBST: Tris-buffered saline Tween 20
TEER: Transendothelial electrical resistance
TRITC: Tetraethyl rhodamine isothiocyanate
UFH: Unfractionated heparin
VEGF: Vascular endothelial growth factor
W/D: Wet/dry

## References

[1] Evans CE, Zhao YY (2017). Impact of thrombosis on pulmonary endothelial injury and repair following sepsis. American journal of physiology lung cellular and molecular. Physiology.

[2] Mikkelsen ME, Shah CV, Meyer NJ, Gaieski DF, Lyon S, Miltiades AN (2013). The epidemiology of acute respiratory distress syndrome in patients presenting to the emergency department with severe sepsis. Shock.

[3] Ware LB (2006). Pathophysiology of acute lung injury and the acute respiratory distress syndrome. Seminars in respiratory and critical care medicine.

[4] Bianchi AM, Reboredo MM, Lucinda LM, Reis FF, Silva MV, Rabelo MA (2016). The effects of prone position ventilation on experimental mild acute lung injury induced by intraperitoneal lipopolysaccharide injection in rats. Lung.

[5] Bogatcheva NV, Verin AD (2008). The role of cytoskeleton in the regulation of vascular endothelial barrier function. Microvasc Res.

[6] Kasa A, Csortos C, Verin AD (2015). Cytoskeletal mechanisms regulating vascular endothelial barrier function in response to acute lung injury. Tissue barriers.

[7] Mikelis CM, Simaan M, Ando K, Fukuhara S, Sakurai A, Amornphimoltham P (2015). RhoA and ROCK mediate histamine-induced vascular leakage and anaphylactic shock. Nat Commun.

[8] Li Y, Wu Y, Wang Z, Zhang XH, Wu WK (2010). Fasudil attenuates lipopolysaccharide-induced acute lung injury in mice through the rho/rho kinase pathway. Medical science monitor : international medical journal of experimental and clinical research.

[9] Krendel M, Zenke FT, Bokoch GM (2002). Nucleotide exchange factor GEF-H1 mediates cross-talk between microtubules and the actin cytoskeleton. Nat Cell Biol.

[10] Kratzer E, Tian Y, Sarich N, Wu T, Meliton A, Leff A (2012). Oxidative stress contributes to lung injury and barrier dysfunction via microtubule destabilization. Am J Respir Cell Mol Biol.

[11] Birukova AA, Birukov KG, Smurova K, Adyshev D, Kaibuchi K, Alieva I (2004). Novel role of microtubules in thrombin-induced endothelial barrier dysfunction. FASEB J.

[12] Mousavi S, Moradi M, Khorshidahmad T, Motamedi M (2015). Anti-inflammatory effects of heparin and its derivatives: a systematic review. Adv Pharmacol Sci.

[13] Li X, Zheng Z, Mao Y, Ma X (2012). Unfractionated heparin promotes LPS-induced endothelial barrier dysfunction: a preliminary study on the roles of angiopoietin/Tie2 axis. Thromb Res.

[14] Han J, Ding R, Zhao D, Zhang Z, Ma X (2013). Unfractionated heparin attenuates lung vascular leak in a mouse model of sepsis: role of RhoA/rho kinase pathway. Thromb Res.

[15] Schieven GL (2005). The biology of p38 kinase: a central role in inflammation. Curr Top Med Chem.

[16] Chu ZG, Zhang JP, Song HP, Hu JY, Zhang Q, Xiang F (2010). p38 MAP kinase mediates burn serum-induced endothelial barrier dysfunction: involvement of F-actin rearrangement and L-caldesmon phosphorylation. Shock.

[17] Li X, Zheng Z, Li X, Ma X (2012). Unfractionated heparin inhibits lipopolysaccharide-induced inflammatory response through blocking p38 MAPK and NF-kappaB activation on endothelial cell. Cytokine.

[18] Liu Z, Wang L, Dong Z, Pan J, Zhu H, Zhang Z (2015). Heparin inhibits lipopolysaccharide-induced inflammation via inducing caveolin-1 and activating the p38/mitogen-activated protein kinase pathway in murine peritoneal macrophages. Mol Med Rep.

[19] Ding R, Zhao D, Guo R, Zhang Z, Ma X (2011). Treatment with unfractionated heparin attenuates coagulation and inflammation in endotoxemic mice. Thromb Res.

[20] Monaghan-Benson E, Wittchen ES (2011). In vitro analyses of endothelial cell permeability. Methods Mol. Biol.

[21] Putnam AJ, Cunningham JJ, Dennis RG, Linderman JJ, Mooney DJ (1998). Microtubule assembly is regulated by externally applied strain in cultured smooth muscle cells. J Cell Sci.

[22] Birukova AA, Fu P, Xing J, Yakubov B, Cokic I, Birukov KG (2010). Mechanotransduction by GEF-H1 as a novel mechanism of ventilator-induced vascular endothelial permeability. Am. J. Physiol. Lung Cell. Mol. Physiol.

[23] Janke C, Bulinski JC (2011). Post-translational regulation of the microtubule cytoskeleton: mechanisms and functions. Nat Rev Mol Cell Biol.

[24] Zarychanski R, Abou-Setta AM, Kanji S, Turgeon AF, Kumar A, Houston DS (2015). The efficacy and safety of heparin in patients with sepsis: a systematic review and metaanalysis. Crit Care Med.

[25] Wang C, Chi C, Guo L, Wang X, Guo L, Sun J (2014). Heparin therapy reduces 28-day mortality in adult severe sepsis patients: a systematic review and meta-analysis. Crit care.

[26] Li X, Li Z, Zheng Z, Liu Y, Ma X (2013). Unfractionated heparin ameliorates lipopolysaccharide-induced lung inflammation by downregulating nuclear factor-kappaB signaling pathway. Inflammation.

[27] Rizzo AN, Letsiou E, Sammani S, Esquinca A, Garcia JG, Dudek SM (2014). Abstract 13333: Imatinib attenuates LPS-induced inflammation and vascular leakage in clinically relevant murine models of acute lung injury. Circulation.

[28] Shen W, Gan J, Xu S, Jiang G, Wu H (2009). Penehyclidine hydrochloride attenuates LPS-induced acute lung injury involvement of NF-kappaB pathway. Pharmacol Res.

[29] Abdelmageed ME, El-Awady MS, Suddek GM (2016). Apocynin ameliorates endotoxin-induced acute lung injury in rats. Int Immunopharmacol.

[30] Kawasaki M, Kuwano K, Hagimoto N, Matsuba T, Kunitake R, Tanaka T (2000). Protection from lethal apoptosis in lipopolysaccharide-induced acute lung injury in mice by a caspase inhibitor. Am J Pathol.

[31] Wang L, Wang T, Li H, Liu Q, Zhang Z, Xie W (2016). Receptor interacting protein 3-mediated necroptosis promotes lipopolysaccharide-induced inflammation and acute respiratory distress syndrome in mice. PLoS One.

[32] Wang ZY, Wu SN, Zhu ZZ, Yang BX, Zhu X (2013). Inhaled unfractionated heparin improves abnormalities of alveolar coagulation, fibrinolysis and inflammation in endotoxemia-induced lung injury rats. Chin Med J.

[33] Jesmin S, Zaedi S, Islam AM, Sultana SN, Iwashima Y, Wada T (2012). Time-dependent alterations of VEGF and its signaling molecules in acute lung injury in a rat model of sepsis. Inflammation.

[34] Opal SM, van der Poll T (2015). Endothelial barrier dysfunction in septic shock. J Intern Med.

[35] Chang YC, Nalbant P, Birkenfeld J, Chang ZF, Bokoch GM (2008). GEF-H1 couples nocodazole-induced microtubule disassembly to cell contractility via RhoA. Mol Biol Cell.

[36] Birukova AA, Birukov KG, Adyshev D, Usatyuk P, Natarajan V, Garcia JG (2005). Involvement of microtubules and Rho pathway in TGF-beta1-induced lung vascular barrier dysfunction. J. Cell. Physiol.

[37] Gorshkov BA, Zemskova MA, Verin AD, Bogatcheva NV (2012). Taxol alleviates 2-methoxyestradiol-induced endothelial permeability. Vasc Pharmacol.

[38] Yu L, Quinn DA, Garg HG, Hales CA (2011). Heparin inhibits pulmonary artery smooth muscle cell proliferation through guanine nucleotide exchange factor-H1/RhoA/rho kinase/p27. Am J Respir Cell Mol Biol.

[39] Redeker V, Levilliers N, Schmitter JM, Le Caer JP, Rossier J, Adoutte A (1994). Polyglycylation of tubulin: a posttranslational modification in axonemal microtubules. Science.

[40] Bounoutas A, Kratz J, Emtage L, Ma C, Nguyen KC, Chalfie M (2011). Microtubule depolymerization in Caenorhabditis elegans touch receptor neurons reduces gene expression through a p38 MAPK pathway. Proc Natl Acad Sci.

[41] Bogatcheva NV, Adyshev D, Mambetsariev B, Moldobaeva N, Verin AD (2007). Involvement of microtubules, p38, and rho kinases pathway in 2-methoxyestradiol-induced lung vascular barrier dysfunction. Am. J. Physiol. Lung Cell. Mol. Physiol.

[42] Armstrong SC, Delacey M, Ganote CE (1999). Phosphorylation state of hsp27 and p38 MAPK during preconditioning and protein phosphatase inhibitor protection of rabbit cardiomyocytes. J Mol Cell Cardiol.

[43] Pan YX, Chen KF, Lin YX, Wu W, Zhou XM, Zhang XS (2013). Intracisternal administration of SB203580, a p38 mitogen-activated protein kinase inhibitor, attenuates cerebral vasospasm via inhibition of tumor-necrosis factor-alpha. J. Clin. Neurosci.

[44] Sarker KP, Nakata M, Kitajima I, Nakajima T, Maruyama I (2000). Inhibition of caspase-3 activation by SB 203580, p38 mitogen-activated protein kinase inhibitor in nitric oxide-induced apoptosis of PC-12 cells. J Mol Neurosci.

[45] Sreekanth GP, Chuncharunee A, Sirimontaporn A, Panaampon J, Noisakran S, Yenchitsomanus PT (2016). SB203580 modulates p38 MAPK signaling and dengue virus-induced liver injury by reducing MAPKAPK2, HSP27, and ATF2 phosphorylation. PLoS One.

[46] Zhou Z, Guo F, Yi L, Tang J, Dou Y, Huan J (2015). The p38/mitogen-activated protein kinase pathway is implicated in lipopolysaccharide-induced microtubule depolymerization via up-regulation of microtubule-associated protein 4 phosphorylation in human vascular endothelium. Surgery.

[47] Liang Y, Li X, Zhang X, Li Z, Wang L, Sun Y (2014). Elevated levels of plasma TNF-alpha are associated with microvascular endothelial dysfunction in patients with sepsis through activating the NF-kappaB and p38 mitogen-activated protein kinase in endothelial cells. Shock.

[48] Bannon PG, Kim MJ, Dean RT, Dawes J (1995). Augmentation of vascular endothelial barrier function by heparin and low molecular weight heparin. Thromb Haemost.

[49] Young E, Venner T, Ribau J, Shaughnessy S, Hirsh J, Podor TJ (1999). The binding of unfractionated heparin and low molecular weight heparin to thrombin-activated human endothelial cells. Thromb Res.

[50] Martinez-Sales V, Vila V, Reganon E, Oms JG, Aznar J (2003). Effect of unfractionated heparin and a low molecular weight heparin (enoxaparin) on coagulant activity of cultured human endothelial cells. Haematologica.

[51] Vignoli A, Marchetti M, Balducci D, Barbui T, Falanga A (2006). Differential effect of the low-molecular-weight heparin, dalteparin, and unfractionated heparin on microvascular endothelial cell hemostatic properties. Haematologica.

[52] Kosoff R, Chow HY, Radu M, Chernoff J (2013). Pak2 kinase restrains mast cell FcepsilonRI receptor signaling through modulation of rho protein guanine nucleotide exchange factor (GEF) activity. J Biol Chem.

[53] Birukova AA, Smurova K, Birukov KG, Usatyuk P, Liu F, Kaibuchi K (2010). Microtubule disassembly induces cytoskeletal remodeling and lung vascular barrier dysfunction: role of rho-dependent mechanisms. J Cell Physiol.

[54] Holcomb JB (2011). A novel and potentially unifying mechanism for shock induced early coagulopathy. Ann Surg.

[55] Yini S, Heng Z, Xin A, Xiaochun M (2015). Effect of unfractionated heparin on endothelial glycocalyx in a septic shock model. Acta Anaesthesiol Scand.

[56] Chen S, He Y, Hu Z, Lu S, Yin X, Ma X (2017). Heparanase mediates intestinal inflammation and injury in a mouse model of Sepsis. J. Histochem. Cytochem.

[57] Palumbo S, Shin YJ, Ahmad K, Desai AA, Quijada H, Mohamed M (2017). Dysregulated Nox4 ubiquitination contributes to redox imbalance and age-related severity of acute lung injury. Am. J. Physiol. Lung Cell. Mol. Physiol.

